# IL-2 and IL-15 dependent thymic development of Foxp3-expressing regulatory T lymphocytes

**DOI:** 10.1007/s13238-017-0425-3

**Published:** 2017-05-24

**Authors:** Cécile Apert, Paola Romagnoli, Joost P. M. van Meerwijk

**Affiliations:** CPTP, Université de Toulouse, CNRS, Inserm, UPS, Toulouse, France

**Keywords:** regulatory T cells, thymus, differentiation, IL-2, IL-15

## Abstract

Immunosuppressive regulatory T lymphocytes (Treg) expressing the transcription factor Foxp3 play a vital role in the maintenance of tolerance of the immune-system to self and innocuous non-self. Most Treg that are critical for the maintenance of tolerance to self, develop as an independent T-cell lineage from common T cell precursors in the thymus. In this organ, their differentiation requires signals from the T cell receptor for antigen, from co-stimulatory molecules, as well as from cytokine-receptors. Here we focus on the cytokines implicated in thymic development of Treg, with a particular emphasis on the roles of interleukin-2 (IL-2) and IL-15. The more recently appreciated involvement of TGF-β in thymic Treg development is also briefly discussed. Finally, we discuss how cytokine-dependence of Treg development allows for temporal, quantitative, and potentially qualitative modulation of this process.

## INTRODUCTION

Several populations of T lymphocytes with immunosuppressive activity have been identified to date (Josefowicz et al., 2012a; Kim and Cantor, 2011; Sakaguchi et al., 2010; Shevach, 2011; Vuddamalay and van Meerwijk, 2017; Xu et al., 2016). The most critical population of Treg expresses Foxp3: Humans and mice carrying mutations in the gene encoding this forkhead/winged helix transcription factor develop a rapidly lethal autoimmune and inflammatory pathology (Bennett et al., 2001; Brunkow et al., 2001; Fontenot et al., 2003). Treg therefore prevent the development of autoimmune pathology and chronic inflammation in interfaces with the environment such as the intestines, lungs, and skin.

As other T lymphocytes, activation of Treg requires that they recognize antigenic peptides presented by cell-surface expressed molecules encoded in the major histocompatibility complex (MHC). Autoimmune pathology develops upon activation of autospecific conventional T cells (Tconv) and suppression of these immune-responses involves autospecific Treg (Kieback et al., 2016; Yang et al., 2015). The antigen-specificity of Treg involved in the prevention of chronic inflammatory diseases remains less well defined, but antigens from commensals as well as from infectious agents are most certainly recognized. It therefore appears appealing to postulate that Treg preventing autoimmune pathology develop mostly in the thymus where autospecific T cell precursors can recognize self-antigens presented by thymic stromal cells (Klein and Jovanovic, 2011). It needs to be emphasized, however, that autospecific Treg can also differentiate from Tconv precursors in peripheral lymphoid organs (Sun et al., 2015). The Treg preventing chronic inflammation likely mostly differentiate in peripheral (lymphoid) organs where Tconv involved in immune-responses, and therefore specific for the agents that provoked the inflammation, differentiate into Treg under tolerogenic conditions (Bilate and Lafaille, 2012; Josefowicz et al., 2012b). Nevertheless, colonic Treg express T cell receptors for antigen (TCR) that were also found on thymic Treg, suggesting that Treg involved in the prevention of chronic inflammation may develop in the thymus (Cebula et al., 2013). Recent data showing that peripheral Treg recirculate back to the thymus and that they represent a very substantial proportion of thymic Treg in adult mice (Cowan et al., 2016; Thiault et al., 2015), however, urge for a reinvestigation of this issue.

In addition to signals via the TCR, cytokine signaling plays a central role in Treg differentiation in the thymus and the periphery. In this review, we focus on Treg development in the thymus and will discuss TCR- and cytokine receptor-derived signals. We will discuss how and where these distinct signals intersect to drive development of a Treg repertoire which efficiently protects the organism from autoimmune pathology.

## TCR-SIGNALING AND THYMIC TREG-DIFFERENTIATION

Within the thymus two anatomically and functionally distinct compartments can be distinguished; the cortex and the medulla. Hematopoietic precursors enter the thymus at the cortico-medullary junction. During their differentiation, thymocytes will first migrate into the cortex, then back to the medulla before leaving the thymus at the cortico-medullary junction (Love and Bhandoola, 2011). Thymocytes acquire the expression of the heterodimeric TCRαβ. The genes encoding the TCR undergo random somatic rearrangements that allow for the generation of a wide repertoire of TCR capable of recognizing a vast variety of rapidly evolving infectious agents. Distinct TCR-based selection processes ensure that only functional and harmless T cells leave the thymus (Kurd and Robey, 2016; Lucas et al., 2016; Ohigashi et al., 2016).

In the thymic cortex, only thymocytes bearing a TCR with a certain affinity for peptide/MHC complexes presented by cortical thymic epithelial cells (cTEC) survive. This process, called positive selection, ensures the selection of developing T cells expressing a “functional” TCR and contributes to the generation of a repertoire of T cells expressing TCR that can recognize antigens in the context of self MHC-molecules. Positively selected thymocytes rapidly upregulate the chemokine receptor CCR4 which attracts these cells to CCL17 and CCL22-producing, Sirpα^+^ dendritic cells (DC) and to the medulla (Hu et al., 2015). Too high affinity interactions of the TCR with peptide/MHC complexes, expressed by presumably DC, lead to a first wave of negative selection of autospecific and therefore dangerous T cell-precursors (McCaughtry et al., 2008; Stritesky et al., 2013). Thus deleted T cell-precursors are likely specific for ubiquitously expressed and potentially for epithelial antigens. T lymphocytes that developed in a thymus in which MHC molecules were exclusively expressed by cTEC are strongly autoreactive, suggesting that cTEC contribute little, if at all, to negative selection (Capone et al., 2001; Laufer et al., 1996). Interestingly, high affinity interactions in the cortex may also lead to the differentiation of Treg (Liston et al., 2008; Ribot et al., 2007), but Treg-differentiation in the cortex remains a little-studied process. Thymocytes surviving cortical selection upregulate the chemokine-receptor CCR7 and migrate to the medulla in response to CCL19 and CCL21 produced by medullary thymic epithelial cells (mTEC) (Ueno et al., 2004).

In the thymic medulla, thymocytes interact via their TCR with peptide/MHC complexes presented by mTEC, DC, and B lymphocytes. High avidity interactions of thymocytes with medullary stromal cells can lead to their negative selection through induction of apoptosis (“deletion”) or clonal anergy (Kyewski and Klein, 2006). High avidity interactions of developing T cells with all cited types of thymic stromal cells also lead to the differentiation of Treg (Klein et al., 2009) and the majority of developing Treg is localized in the medulla rather than in the cortex (Thiault et al., 2015). Combined, the processes of negative selection and Treg-differentiation in the thymus play a major role in the establishment of immune-tolerance to self-antigens.

It has therefore been clearly established that high avidity interactions of the developing T cells with thymic stromal cells can lead to two quite distinct outcomes: negative selection and differentiation of Treg. It remains less clear what tips the balance between these two fates. The mere affinity of the TCR with peptide/MHC complexes is not responsible. In mice expressing a single TCR specific for a non-self-antigen, no Treg develop. When the recognized antigen is introduced as a transgene, deletion as well as Treg-differentiation take place (Klein and Jovanovic, 2011). Other factors must therefore be involved. The observation that the distinct medullary stromal cell-types induce Treg-differentiation at distinct doses of antigenic peptide *in vitro*, suggests that the stromal cell with which thymocytes interact *in vivo* may in part determine the choice between deletion and Treg-differentiation (Wirnsberger et al., 2009). This may be due to the distinct surface expression levels of ligands for e.g. CD28, CD27 or other members of the TNF-receptor superfamily, or other molecules involved in deletion and Treg-differentiation (Coquet et al., 2013; Mahmud et al., 2014; Tai et al., 2005; Tang et al., 2003). However, even in experimental systems in which agonist peptide/MHC ligand was presumably exclusively presented by a single stromal cell-type, i.e., mTEC, deletion as well as Treg-differentiation were observed (Aschenbrenner et al., 2007). At least two explanations can be proposed. First, Treg-lineage commitment may take place independently of the thymocyte’s TCR (Pennington et al., 2006) and distinct selection criteria for Tconv and Treg precursors determine development of these two populations. Second, heterogeneity among mTEC (and potentially the other stromal cell-types) (Brennecke et al., 2015; Meredith et al., 2015) may be involved. These issues would merit further investigation.

## EPIGENETIC MODIFICATIONS AND THYMIC DEVELOPMENT OF TREG

Epigenetic gene regulation, such as DNA methylation and histone modifications, is implicated in lineage specification and maintenance. Several groups have demonstrated that DNA demethylation at conserved non-coding sequence within the *Foxp3* locus ensures the stability of its expression in thymic derived Treg (Floess et al., 2007; Kim and Leonard, 2007; Zheng et al., 2010). It was shown that DNA methylation is lost during the last (i.e., Foxp3-expressing) stages of thymic Treg-development through oxidation of 5-methylcytosine and other intermediates in the demethylation process. It was suggested that two enzymes, TET2 and TET3, initiate this reaction (Toker et al., 2013). Indeed, in *Tet2*/*Tet3* double deficient mice, in which regulatory regions remain methylated, Foxp3 expression is unstable and Treg lose their suppressive functions (Yue et al., 2016). Interestingly, Treg-specific demethylated regions (TSDRs) are also found in other genes encoding for factors essential for Treg function, such as CD25, CTLA-4, Eos, and GITR (Ohkura et al., 2012). While TCR signaling is required for demethylation of TSDRs, *Foxp3* gene expression is dispensable. These data indicate that to establish Treg lineage two independent but complementary molecular mechanisms are in play: *Foxp3* gene expression and epigenetic changes (Ohkura et al., 2012). Establishment of a Treg epigenetic landscape may therefore precede and promote *Foxp3* gene expression. CpG demethylation (or initiation of this process) in the TSDR or CNS2 of the *Foxp3* gene strictly correlated with expression of this gene, yielding little insight into this question (Toker et al., 2013; Yue et al., 2016). However, the recently described binding of a global chromatin organizer, Satb1, to another regulatory region of the *Foxp3* locus (CNS0) in immature CD4/CD8 double positive thymocytes and its requirement for Treg development suggest that early epigenetic modifications control the expression of Foxp3 and Treg signature genes (Kitagawa et al., 2017). How the expression and activity of Satb1 are regulated remains to be determined.

## INVOLVEMENT OF IL-2 AND IL-15 IN TREG DIFFERENTIATION IN THE THYMUS

Early studies with mice genetically deficient in production of the “T cell growth factor” IL-2 or expression of its receptor surprisingly showed that these animals developed severe autoimmune pathology instead of immunodeficiency (Sadlack et al., 1995; Suzuki et al., 1995; Willerford et al., 1995). Initially, defects in IL-2 dependent activation induced cell-death (AICD) of autoreactive T cells were suspected. However, complementation of mice deficient in IL-2 or its receptor with WT Treg prevented pathology (Suzuki et al., 1999; Wolf et al., 2001). The latter data indicated that a lack of Treg or Treg-functional capacity was responsible for the lymphoproliferation and lethal autoimmune pathology in mutant mice. It was later appreciated that IL-2 plays a major role in Treg homeostasis. The role of IL-2 in the differentiation of Treg from Tconv precursors in peripheral lymphoid organs (and potentially in tissues) and in survival and function of mature Treg has recently been discussed (Chinen et al., 2016) and is beyond the scope of this review.

One of the earliest indications that IL-2 may play a role in the thymic development of Treg came from studies by Malek and colleagues showing that mice in which the IL-2Rβ was exclusively expressed by developing thymocytes, survived substantially longer than IL-2Rβ-deficient animals (Malek et al., 2000). Later studies showed that substantially reduced proportions of mature CD4^+^CD25^+^ regulatory thymocytes developed in IL-2Rβ-deficient mice and that differentiation of Treg with *in vitro* immunosuppressive activity was fully restored in mice in which IL-2Rβ expression was restricted to thymocytes (Burchill et al., 2007; Fontenot et al., 2005; Malek et al., 2002; Soper et al., 2007; Vang et al., 2008).

Several other studies further addressed the role for IL-2 in Treg development in the thymus using distinct mutant mice. Rudensky and colleagues found approximately half the number of Treg in the thymus of IL-2-deficient as compared to that of WT mice (Fontenot et al., 2005). However, other studies found practically normal numbers of Treg in IL-2 deficient mice (Burchill et al., 2007; D’Cruz and Klein, 2005). Similarly discordant results were found in studies with mice deficient in the IL-2Rα chain (D’Cruz and Klein, 2005; Fontenot et al., 2005; Soper et al., 2007). It therefore appears that IL-2 may play a role in Treg development, but that it is not strictly required.

How then to explain the substantially reduced numbers of Treg in the thymus of IL-2Rβ deficient mice, a very consistent observation? The IL-2Rβ chain is shared between the receptors for IL-2 and for IL-15. The latter cytokine may therefore play an important role in Treg development. Farrar and colleagues found approximately half the number of Treg in the thymus of IL-15 deficient mice as compared to WT animals (Burchill et al., 2007). In IL-2 and IL-15 doubly deficient thymuses, they found as few Treg as in the thymus of IL2Rβ-deficient mice. However, Ziegler and colleagues found normal proportions of Treg in the thymus of mice lacking expression of the IL-15Rα chain, which is only involved in signalling via the receptor for IL-15 (Soper et al., 2007). It therefore appears likely that IL-15 is involved in Treg development in the thymus but, again, that it is not absolutely required.

Therefore, whereas in mice lacking expression of both IL-2 and IL-15 or both of their receptors substantially reduced numbers of Treg were consistently found in the thymus, in mice deficient for one or the other cytokine or receptor discordant results were obtained. These data therefore clearly demonstrate that the cytokines IL-2 and IL-15 play important roles in the thymic development of Treg. What then explains the discordant results when only one of the two cytokines or receptors is missing? In transgenic mice in which the green-fluorescent protein is expressed under control of the *Rag2* promoter (“Rag2-GFP mice”), newly developing T cells are GFP^+^ and cells that are “older” do no longer express GFP (Yu et al., 1999). Using Rag2-GFP mice, it was observed that only a small proportion of thymic Treg are actually newly developing cells (Cuss and Green, 2012; McCaughtry et al., 2007; Thiault et al., 2015). It was therefore important to assess the roles for IL-2 and IL-15 in *de novo* development of Treg. Seddon and colleagues used a particularly original approach to study *de novo* T cell development in which the developmental blockade of T cells in ZAP-70 deficient mice can be lifted by inducing ZAP70 expression using a TetOn transgenic system (Marshall et al., 2014). It was thus unambiguously shown that in absence of signaling through the receptors for IL-2 or of IL-15 Treg development was severely hampered. These results strongly indicate important and non-redundant roles for IL-2 and IL-15 in Treg development in the murine thymus. Importantly, using *in vitro* culture systems, Sousa and colleagues demonstrated quantitatively important and non-redundant roles for IL-2 and IL-15 in the development of Foxp3-expressing human Treg from immature thymic precursors (Caramalho et al., 2015).

## THE MECHANISMS BY WHICH IL-2 AND IL-15 CONTROL TREG-DEVELOPMENT IN THE THYMUS

To investigate how IL-2 and IL-15 impinge on Treg development in the thymus, it was important to determine at what stage these cytokines act. Treg and Tconv develop from common precursors. The CD4^+^CD8^−^TCR^high^ (“CD4SP”) stage of development is apparently the last one shared between Treg and Tconv. The CD4SP stage can be further dissected into four subpopulations using expression of CD25 and of Foxp3 as criterion. In cultures of electronically sorted CD4SP CD25^−^Foxp3^−^ thymocytes, no Foxp3^+^ cells develop and the addition of IL-2 or IL-15 does not change this result (Lio and Hsieh, 2008). By contrast, some CD25^+^Foxp3^−^ cells expressed Foxp3 upon *in vitro* culture and this Treg differentiation was greatly increased by addition of IL-2 or IL-15 (Lio and Hsieh, 2008; Tai et al., 2013; Vang et al., 2008). Similar results were obtained using human CD4SP CD25^+^ CD127^+^ (in large majority Foxp3^−^) thymocytes (Caramalho et al., 2015). These results suggested that CD4SP CD25^+^Foxp3^−^ thymocytes, which develop independently of IL-2 and IL-15 (Marshall et al., 2014), are the Treg-precursors that require IL-2 or IL-15 for their final steps of differentiation. However, in absence of signaling through the receptor for IL-15 (but not IL-2) the development of another CD4SP subpopulation, CD25^−^Foxp3^+^ cells, was found to be reduced (Marshall et al., 2014). Upon *in vitro* culture of WT CD4SP CD25^−^Foxp3^+^ thymocytes in presence of IL-2, these cells become CD25^+^Foxp3^+^ Treg (Marshall et al., 2014; Tai et al., 2013).

These and other data indicate that CD4SP CD25^+^Foxp3^+^ Treg can develop via two distinct pathways. In the first pathway, TCR-signaling allows CD4SP CD25^−^Foxp3^−^ thymocytes to become CD25^+^Foxp3^−^ cells which, when stimulated through their receptors for IL-2 or IL-15, will become CD25^+^Foxp3^+^ Treg. In the second pathway, signaling through the TCR and the IL-15 receptor allows CD4SP CD25^−^Foxp3^−^ thymocytes to become CD25^−^Foxp3^+^ cells which, when stimulated through their receptor for IL-2, will become CD25^+^Foxp3^+^ Treg. Interestingly, experiments in which a Nur77-GFP reporter indicated the affinity of TCR-peptide/MHC interactions suggested that the development of CD4SP CD25^+^Foxp3^−^ precursors involves higher affinity interactions with stromal cells than differentiation of CD25^−^Foxp3^+^ precursors (Marshall et al., 2014).

In both proposed developmental pathways, the cytokines IL-2 and IL-15 therefore appear to induce expression of Foxp3. Signaling downstream of the receptors for IL-2 and IL-15 involves the JAK3-STAT5 pathway. Consistent with the roles for these cytokines in Treg differentiation in the thymus, in mice with a T cell-specific deficiency in STAT5 expression a severe defect in Treg development was observed (Burchill et al., 2007). Moreover, transgenic expression of constitutively active STAT5 rescued Treg development in IL-2Rβ-deficient mice (Burchill et al., 2003; Burchill et al., 2007). The *Foxp3* gene contains several conserved potential STAT5 binding sites, some of which demonstrated binding of STAT5 in Treg but not Tconv (Burchill et al., 2007; Yao et al., 2007). It therefore appears that signaling through the receptors for IL-2 and IL-15 induces, via STAT5 activation, expression of Foxp3.

An alternative but not mutually exclusive explanation for the action of IL-2 (and potentially IL-15) was reported by Singer and colleagues. They showed that Foxp3 expression promotes cell-death by inducing expression of pro-apoptotic (e.g., PUMA and BIM) and repressing pro-survival proteins (e.g., Bcl-2) (Tai et al., 2013). *In vitro* experiments showed that IL-2 protected Foxp3^+^ but not Foxp3^−^ CD4SP cells from apoptosis by increasing the expression of Bcl-2, which was mediated by STAT5. Though intriguing, these results will need to be reconciled with the observation that transgenic expression of Foxp3 restored Treg development and prevented autoimmune pathology in IL-2Rβ deficient mice (Soper et al., 2007).

IL-2 and IL-15 appear not implicated in the induction of the demethylation of the regulatory region of the *Foxp3* locus of Treg precursors. Compared to the strong TSDR demethylation observed in CD25^+^Foxp3^−^ cells stimulated via their TCR in the presence of IL-2, precursors cultured *in vitro* with IL-2 or IL-15 alone display only a minor TSDR demethylation (8%–14%) (Toker et al., 2013).

## THE CELLULAR ORIGINS OF THE IL-2 AND IL-15 INVOLVED IN TREG DEVELOPMENT

Identification of the cellular sources of the IL-2 and IL-15 involved in Treg differentiation may reveal important regulatory mechanisms of this process (*cf. infra*). In hematopoietic chimeras in which only radioresistant stromal cells carried an invalidating mutation in the *Il2* gene, normal numbers of Treg developed, strongly suggesting that the IL-2 involved in Treg development is produced by (radiosensitive) hematopoietic cells (Marshall et al., 2014). Probably the most obvious candidates for the cells producing IL-2 in the thymus are (developing) T cells. IL-2 would then potentially act in an autocrine manner to stimulate Treg differentiation. In mixed hematopoietic chimeras, *Il2*-mutant thymocytes can efficiently differentiate into Treg when other hematopoietic cells can produce IL-2 (Tai et al., 2005), suggesting that an autocrine loop does not play a major role. Robey and colleagues showed that IL-2 derived from DC added to *in vitro* thymic slice cultures could drive Treg development (Weist et al., 2015). In the human thymus, IL-2 protein was found in the cortex and the medulla, and the *Il2* gene appeared expressed mostly by mature T cells (Caramalho et al., 2015). No evidence was found for IL-2-production by human thymic dendritic cells. It will now be important to identify the precise thymic cell-type(s) that produce(s) the IL-2 involved in Treg differentiation using conditional *Il2* KO mice (Popmihajlov et al., 2012) bred to mice expressing the Cre-recombinase under control of appropriate promoters, e.g., those controlling the genes encoding CD11c, CD4, or p56^lck^.

The identity of the thymic population producing the IL-15 involved in Treg development is probably clearer, at least in the mouse. In hematopoietic chimeras in which radioresistant cells could not present, via their IL-15 receptor α-chain, IL-15 to developing thymocytes, strongly reduced proportions of Treg developed (Marshall et al., 2014). The thymic development of iNKT cells also requires IL-15, which is presented by radioresistant stromal (but not dendritic) cells (Castillo et al., 2010). Using reporter mice, Ikuta and co-workers showed that, in the thymus, IL-15 is produced mainly by mTEC and, at lower levels, by some pericytes and blood endothelial cells (Cui et al., 2014). Flow-cytometry analysis identified mTEC expressing high levels of MHC class II (“mTEC^high^”) as the main IL-15 producing stromal cell-type in the thymus. By contrast, dendritic cells and macrophages did not produce IL-15. Lefrançois and co-workers demonstrated, using reporter mice, that very immature (and therefore cortical) thymocytes can produce high levels of IL-15 and that these cells rapidly lose this capacity during T cell development (Colpitts et al., 2013). However, given that Treg mainly develop in the thymic medulla suggests that this source of IL-15 is irrelevant to Treg differentiation. Combined, the published data strongly indicate that the IL-15 involved in Treg development in the murine thymus is produced and presented by mTEC^high^. Intriguingly, these stromal cells ectopically express tissue-antigens known to be involved in selection of the Treg’s TCR-repertoire (Aschenbrenner et al., 2007; Malchow et al., 2013; Takaba et al., 2015; Yang et al., 2015). Also in the human thymus, IL-15 was mainly found in the medulla. As in mice, mTEC expressed the *Il15* gene. However, also B cells, monocytes/macrophages, and NK cells produced this cytokine (Caramalho et al., 2015). Given that B lymphocytes can induce T cell-tolerance in the mouse thymus, the latter observation may prove of importance (Yamano et al., 2015).

The distinct signals involved in T cell differentiation in peripheral tissue are not necessarily derived from the same cell. Thus, differentiation of Th2 cells, specialized in the defense against large extracellular parasites such as helminthes, requires TCR triggering through interactions with peptide/MHC complexes, presented by dendritic cells, and the Th2-polarizing cytokine IL-4, which can be derived from type 2 innate lymphoid cells (Pelly et al., 2016). Signals involved in T cell development and activation do not need to be delivered all at once neither and can be accumulated through multiple sequential interactions with different antigen-presenting cells in a multistep process (Khailaie et al., 2014; Kisielow and Miazek, 1995; Valitutti et al., 1995; Wilkinson et al., 1995). Similar mechanisms may act within the thymus to drive Treg differentiation. In the “two-step model” for Treg differentiation in the thymus proposed by Hsieh and colleagues, the first step involves TCR-triggering which leads to upregulation of the high-affinity receptor for IL-2. The second step involves engagement of the latter receptor by IL-2 which, in the experimental setup used by these authors, was not derived from the same stromal cell (Lio and Hsieh, 2008). Using biphoton microscope analysis of explanted thymic tissue, Robey and colleagues demonstrated that thymocytes migrate very rapidly during their development, which is consistent with the notion that involved stimuli may be derived from distinct stromal cells (Le Borgne et al., 2009). On the other hand, in order to drive Treg differentiation in explanted WT thymic tissue, the dendritic cells that presented the peptide/MHC complexes involved in TCR triggering, that were dropped onto the cultures, also needed to produce IL-2 (Weist et al., 2015). It remains therefore unclear if TCR and IL-2-receptor engagements take place simultaneously or sequentially, and if the involved ligands are expressed by the same or by distinct stromal cells.

## HOW THE REQUIREMENT FOR IL-2 AND IL-15 MAY ALLOW MODULATION OF THE GENERATION OF THE TREG REPERTOIRE

The involvement of IL-2 and IL-15 may allow for control of Treg development in the thymus in quantitative and qualitative terms. Transgenic mice expressing constitutively active STAT5 had three- to seven-fold increased numbers of Treg, suggesting that STAT5 activation (through the receptors for IL-2 or IL-15) is a limiting factor in Treg differentiation in the thymus (Burchill et al., 2008; Burchill et al., 2007). In mutant mice lacking the pro-apoptotic proteins Puma and Bim or expressing anti-apoptotic Bcl-2, substantially increased numbers of Treg were found in the thymus, showing that the large majority of developing Treg normally die (Tai et al., 2013). In mixed hematopoietic chimeras in which growing numbers of Treg precursors expressed the common cytokine receptor γ-chain (“γ_c_”, a constituent of, among others, the receptors for IL-2 and IL-15), the numbers of thymic Treg saturated at approximately 10^6^ cells, suggesting that availability of IL-2 and IL-15 quantitatively controls Treg development (Tai et al., 2013). Using cultures of explanted thymic tissue, Robey and colleagues showed that thymic Treg limited the differentiation of new Treg by competing for IL-2 (Weist et al., 2015). In *in vitro* cultures of human thymocytes, addition of IL-2 or IL-15 substantially increased the development of Treg (Caramalho et al., 2015). Interestingly, also in TCR-ligand doubly transgenic mice, the absolute numbers of Treg found in the thymus were well below the numbers expected if all autospecific T cell-precursors had differentiated into Treg (reviewed in ref. Romagnoli et al., 2008), but the potential involvement of limiting availability of cytokines has, to our knowledge, not been demonstrated.

It therefore appears that the availability of IL-2 (and potentially IL-15) may be a limiting factor in Treg development. IL-2 thus appears to quantitatively regulate the numbers of Treg developing in the thymus. Importantly, the availability of IL-2 appears to diminish with age. T cells activated in the periphery during immune responses can migrate back to the thymus (Hale and Fink, 2009) and Treg are not an exception to this phenomenon. Probably because of their repertoire enriched in autospecific cells and the resulting massive activation of peripheral Treg (Fisson et al., 2003; Hsieh et al., 2004; Romagnoli et al., 2002), a very high proportion of thymic Treg are cells that had re-entered the thymus from the periphery (Cowan et al., 2016; Thiault et al., 2015). We showed that recirculating Treg inhibit the differentiation of their precursors and that they do so by limiting the availability of IL-2. Importantly, the proportion of recirculating Treg among thymocytes grows substantially with age, which results in a strongly fading *de novo* development of Treg (Thiault et al., 2015). Treg recirculation may also be modulated by e.g., infection, stress of other factors, which thus would potentially lead to increased or decreased Treg differentiation in the thymus.

Intriguingly, recirculating Treg appeared to control IL-2-dependent but not IL-2-independent Treg differentiation in the thymus (Thiault et al., 2015). Given that the proportion of recirculating Treg among thymocytes substantially increases with age, IL-2- (but not IL-15-) dependent Treg differentiation will strongly decline. It is tempting to speculate that, with age, Treg differentiation will decreasingly depend on IL-2-producing dendritic cells and progressively on interactions with IL-15-producing mTEC. The potential consequences of this shift on e.g., selection of the TCR-repertoire expressed by developing Treg would merit thorough examination.

## INVOLVEMENT OF OTHER γ_C_ AND RELATED CYTOKINES IN TREG DEVELOPMENT

In mice carrying mutations in the gene encoding the common cytokine receptor γ-chain γ_c_, very low proportions of Treg develop in the thymus (Bayer et al., 2008; Burchill et al., 2008; Burchill et al., 2007; Tai et al., 2013). Since γ_c_ is a constituent of the receptors to the cytokines IL-2, IL-4, IL-7, IL-9, IL-15, and IL-21, “γ_c_ cytokines” other than IL-2 and IL-15 may be involved in Treg development in the thymus. IL-4Rα-deficient mice appear to have normal proportions of thymic Foxp3^+^ Treg (Vang et al., 2008). Similar to IL-2 and IL-15, IL-7 induced the conversion of murine CD4SP CD25^+^Foxp3^−^ precursors into Foxp3^+^ Treg in *in vitro* cultures (Vang et al., 2008). IL-7 also drives Treg differentiation of human non-regulatory thymocytes stimulated in presence of TGF-β, but the relevance of this observation for physiological Treg development in the thymus requires clarification (Caramalho et al., 2015). IL-7, presumably produced by thymic epithelial cells (Shitara et al., 2013), plays a major role in early stages of T-lymphocyte development. Therefore, mice deficient in IL-7 or the IL-7Rα chain have severely reduced thymocyte-counts and, consequently, absolute numbers of thymic Treg (Bayer et al., 2008; von Freeden-Jeffry et al., 1995). However, the proportion of Foxp3^+^ cells among CD4SP thymocytes appeared unaltered (Bayer et al., 2008; Vang et al., 2008). To circumvent the effects of IL-7 on early T cell-development, Ikuta and colleagues generated mice in which the gene encoding the IL-7Rα cahin is deleted at the CD4^+^CD8^+^ stage of thymocyte development. The proportion of Foxp3^+^ Treg among CD4SP thymocytes appeared unaltered in these mice (Tani-ichi et al., 2013). It therefore appears unlikely that IL-7 plays a role in the final stages of Treg differentiation in the thymus. This conclusion is coherent with the observation that mice deficient in IL-7 or its receptor do not develop immunopathology (von Freeden-Jeffry et al., 1995).

To the best of our knowledge, the involvement of the γ_c_ cytokines IL-9 and IL-21 in Treg development in the mouse thymus has not been reported. In humans, IL-9 appears to play a role in T cell-development, but involvement in Treg differentiation was not investigated (De Smedt et al., 2000). *In vitro* stimulation of non-regulatory thymocytes in presence of TGF-β leads to Foxp3^+^ Treg differentiation in presence of IL-2 or IL-15, but not of IL-4 nor of IL-21 (Caramalho et al., 2015). Whereas very convincing evidence has been reported for a key-role for IL-2 and IL-15 in intrathymic Treg differentiation, evidence that other γ_c_ cytokines are involved is therefore currently lacking.

Thymic stromal lymphopoietin (TSLP) is an IL-7-related cytokine, the receptor of which shares the IL-7Rα chain with the receptor for IL-7. It was reported that human Hassall’s corpuscles produce TSLP which activates thymic dendritic cells to upregulate expression of CD80 and CD86. *In vitro*, these dendritic cells then appeared to induce the differentiation of Treg from thymocyte precursors (Hanabuchi et al., 2010; Watanabe et al., 2005). Whereas IL-7 induces the differentiation of murine Treg from CD4SP CD25^+^Foxp3^−^ precursors, TSLP does not (Vang et al., 2008). Deficiency of the IL-7Rα chain, and therefore in signaling through the TSLP-R, leads to strongly reduced thymocyte numbers but not to a specific Treg defect (Bayer et al., 2008). Durum and colleagues argued that Treg development requires IL-7 or TSLP mediated stimulation through IL-7Rα (Mazzucchelli et al., 2008). However, it remains unclear why these authors found normal absolute numbers of Treg in the thymus from mice in which radioresistant thymic stromal cells did not produce IL-7 (cf. ref. Shitara et al., 2013). Whereas in humans TSLP may have an indirect role in the thymic generation of Treg, it appears therefore unlikely that TSLP plays a role in this process in mice.

## INVOLVEMENT OF TGF-β IN TREG DEVELOPMENT IN THE THYMUS

Transforming growth factor-β (TGF-β) plays a central role in the differentiation of conventional T cells into Treg in the periphery (“pTreg”) (Bilate and Lafaille, 2012). Treg that differentiated as an independent lineage in the thymus (“tTreg”) appear different from pTreg, e.g., in the methylation status of their *Foxp3* locus (Floess et al., 2007). Moreover, the Foxp3 locus contains at least one TGF-β response element (i.e., CNS1) (Zheng et al., 2010). Even if CNS1 appears dispensable for Treg development in the thymus, TGF-β may play a role in the thymic differentiation of Treg. This hypothesis was investigated in mice in which TGF-β mediated signaling was perturbed. In TGF-β deficient mice, Treg differentiation in the thymus appeared normal (Fahlen et al., 2005; Marie et al., 2005). Thymocytes deficient in expression of the TGF-βRII also developed normally into Foxp3^+^ Treg (Li et al., 2006; Marie et al., 2006). Nevertheless, TGF-β appears to play an important role in thymic Treg differentiation: In the first week of life of mice in which developing thymocytes do not express the TGF-βRI, hardly any Treg differentiate (Liu et al., 2008). However, these few Treg then appear to be activated to proliferate in an IL-2 dependent manner, which appears to explain the finding by several groups of normal proportions of Treg in adult TGF-β (signaling)-deficient animals. It was subsequently suggested that, rather than favoring Treg-differentiation, TGF-β has an anti-apoptotic effect on Treg (Ouyang et al., 2010). However, mice in which the *Tgfbr1* gene was inactivated in differentiated Foxp3^+^ Treg have normal numbers of these cells in the thymus. The latter finding supports a role for TGF-β in differentiation rather than in survival of Treg (Konkel et al., 2014). Interestingly, the involved TGF-β was apparently produced by thymic macrophages, dendritic cells, and epithelial cells in response to apoptosis of developing thymocytes, which potentially explains why Treg develop later than Tconv during ontogeny (Konkel et al., 2014). Other mechanisms may modulate TGF-β production in the thymus and thus quantitatively influence tTreg differentiation.

**Figure 1:**
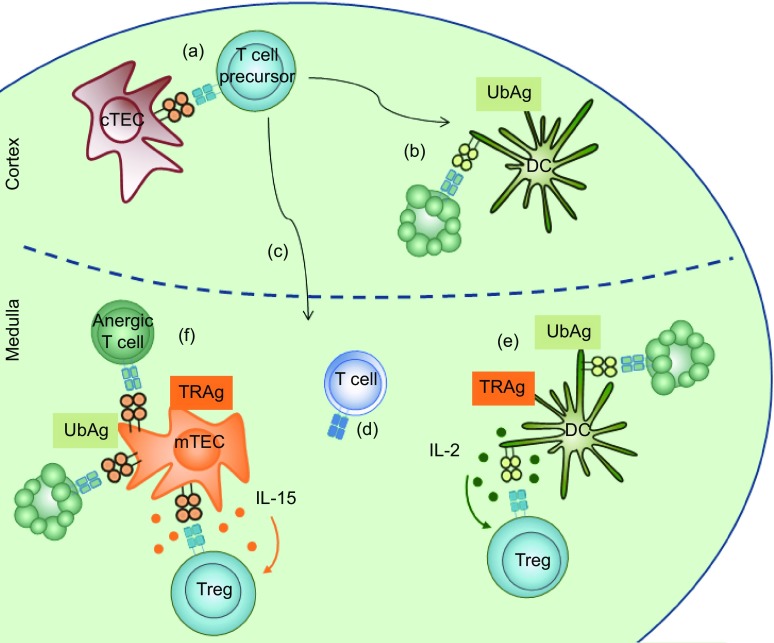
**A model for thymic development and selection of Treg**. Rescue from programmed cell-death of probably common Tconv/Treg precursors requires interaction of their TCR with MHC/peptide complexes expressed on the surface of cortical thymic epithelial cells (cTEC) (a). The thus positively selected precursor-population undergoes a first wave of negative selection (i.e., induction of apoptosis) in the cortex, depleting it of cells specific for the “ubiquitous” antigens (UbAg) presented by cortical dendritic cells (DC) (b). Surviving thymocytes upregulate CCR7 and CCR4 and migrate to the medulla (c) where precursors that recognize MHC/self-peptide complexes with low affinity develop into fully mature Tconv (d). T cell-precursors that recognize MHC/self-peptide complexes with higher affinity have three distinct destinies; become anergic, die by apoptosis, or differentiate into Treg. The latter processes appear to especially concern cells specific for the peripheral-tissue restricted antigens (TRA) expressed by mTEC and presented by mTEC or, upon transfer, by thymic DC. The signals, that determine which of the three distinct outcomes a given autospecific T cell-precursor will adopt, remain incompletely identified, but the IL-2 produced by DC (e) and mTEC-derived IL-15 (f) drive precursors into the Treg lineage

In conclusion, substantial progress has been made in deciphering the roles of IL-2 and IL-15 (and other cytokines) in thymic differentiation of Treg (Fig. 1). The regulation of IL-2 bioavailability by peripheral Treg that recirculate back to the thymus reveals a connection between peripheral immune responses and thymocyte differentiation and offers a new perspective on Treg function. Activated peripheral Treg, therefore, not only control the homeostasis of the immune system in the periphery, but also the development of new Treg in the thymus. Studying the involvement of cytokines in the development of Treg and other T cell-subsets will shed light on modulation, by peripheral immune-responses, of T cell differentiation and TCR-repertoire selection in the thymus.

## References

[CR1] Aschenbrenner K, D’Cruz LM, Vollmann EH, Hinterberger M, Emmerich J, Swee LK, Rolink A, Klein L (2007). Selection of Foxp3(+) regulatory T cells specific for self antigen expressed and presented by Aire(+) medullary thymic epithelial cells. Nature Immunol.

[CR2] Bayer AL, Lee JY, de la Barrera A, Surh CD, Malek TR (2008). A function for IL-7R for CD4+CD25+Foxp3+ T regulatory cells. J Immunol.

[CR3] Bennett CL, Christie J, Ramsdell F, Brunkow ME, Ferguson PJ, Whitesell L, Kelly TE, Saulsbury FT, Chance PF, Ochs HD (2001). The immune dysregulation, polyendocrinopathy, enteropathy, X-linked syndrome (IPEX) is caused by mutations of FOXP3. Nature Genet.

[CR4] Bilate AM, Lafaille JJ (2012). Induced CD4+Foxp3+ regulatory T cells in immune tolerance. Annu Rev Immunol.

[CR5] Brennecke P, Reyes A, Pinto S, Rattay K, Nguyen M, Kuchler R, Huber W, Kyewski B, Steinmetz LM (2015). Single-cell transcriptome analysis reveals coordinated ectopic gene-expression patterns in medullary thymic epithelial cells. Nature Immunol.

[CR6] Brunkow ME, Jeffery EW, Hjerrild KA, Paeper B, Clark LB, Yasayko SA, Wilkinson JE, Galas D, Ziegler SF, Ramsdell F (2001). Disruption of a new forkhead/winged-helix protein, scurfin, results in the fatal lymphoproliferative disorder of the scurfy mouse. Nature Genetics.

[CR7] Burchill MA, Goetz CA, Prlic M, O’Neil JJ, Harmon IR, Bensinger SJ, Turka LA, Brennan P, Jameson SC, Farrar MA (2003). Distinct Effects of STAT5 Activation on CD4+ and CD8+ T Cell Homeostasis: Development of CD4+CD25+ Regulatory T Cells versus CD8+ Memory T Cells. J Immunol.

[CR8] Burchill MA, Yang J, Vogtenhuber C, Blazar BR, Farrar MA (2007). IL-2 receptor beta-dependent STAT5 activation is required for the development of Foxp3+ regulatory T cells. J Immunol.

[CR9] Burchill MA, Yang J, Vang KB, Moon JJ, Chu HH, Lio CW, Vegoe AL, Hsieh CS, Jenkins MK, Farrar MA (2008). Linked T cell receptor and cytokine signaling govern the development of the regulatory T cell repertoire. Immunity.

[CR10] Capone M, Romagnoli P, Beermann F, MacDonald HR, van Meerwijk JPM (2001). Dissociation of thymic positive and negative selection in transgenic mice expressing major histocompatibility complex class I molecules exclusively on thymic cortical epithelial cells. Blood.

[CR11] Caramalho I, Nunes-Silva V, Pires AR, Mota C, Pinto AI, Nunes-Cabaco H, Foxall RB, Sousa AE (2015). Human regulatory T-cell development is dictated by Interleukin-2 and -15 expressed in a non-overlapping pattern in the thymus. J Autoimmun.

[CR12] Castillo EF, Acero LF, Stonier SW, Zhou D, Schluns KS (2010). Thymic and peripheral microenvironments differentially mediate development and maturation of iNKT cells by IL-15 transpresentation. Blood.

[CR13] Cebula A, Seweryn M, Rempala GA, Pabla SS, McIndoe RA, Denning TL, Bry L, Kraj P, Kisielow P, Ignatowicz L (2013). Thymus-derived regulatory T cells contribute to tolerance to commensal microbiota. Nature.

[CR14] Chinen T, Kannan AK, Levine AG, Fan X, Klein U, Zheng Y, Gasteiger G, Feng Y, Fontenot JD, Rudensky AY (2016). An essential role for the IL-2 receptor in Treg cell function. Nat Immunol.

[CR15] Colpitts SL, Stonier SW, Stoklasek TA, Root SH, Aguila HL, Schluns KS, Lefrancois L (2013). Transcriptional regulation of IL-15 expression during hematopoiesis. J Immunol.

[CR16] Coquet JM, Ribot JC, Babala N, Middendorp S, van der Horst G, Xiao Y, Neves JF, Fonseca-Pereira D, Jacobs H, Pennington DJ (2013). Epithelial and dendritic cells in the thymic medulla promote CD4+Foxp3+ regulatory T cell development via the CD27-CD70 pathway. J Exp Med.

[CR17] Cowan JE, McCarthy NI, Anderson G (2016). CCR7 controls thymus recirculation, but not production and emigration, of Foxp3(+) T Cells. Cell reports.

[CR18] Cui G, Hara T, Simmons S, Wagatsuma K, Abe A, Miyachi H, Kitano S, Ishii M, Tani-ichi S, Ikuta K (2014). Characterization of the IL-15 niche in primary and secondary lymphoid organs in vivo. Proc Natl Acad Sci USA.

[CR19] Cuss SM, Green EA (2012). Abrogation of CD40-CD154 signaling impedes the homeostasis of thymic resident regulatory T cells by altering the levels of IL-2, but does not affect regulatory T cell development. J Immunol.

[CR20] D’Cruz LM, Klein L (2005). Development and function of agonist-induced CD25+Foxp3+ regulatory T cells in the absence of interleukin 2 signaling. Nat Immunol.

[CR21] De Smedt M, Verhasselt B, Kerre T, Vanhecke D, Naessens E, Leclercq G, Renauld JC, Van Snick J, Plum J (2000). Signals from the IL-9 receptor are critical for the early stages of human intrathymic T cell development. J Immunol.

[CR22] Fahlen L, Read S, Gorelik L, Hurst SD, Coffman RL, Flavell RA, Powrie F (2005). T cells that cannot respond to TGF-beta escape control by CD4(+)CD25(+) regulatory T cells. J Exp Med.

[CR23] Fisson S, Darrasse-Jeze G, Litvinova E, Septier F, Klatzmann D, Liblau R, Salomon BL (2003). Continuous activation of autoreactive CD4+ CD25+ regulatory T Cells in the steady state. J Exp Med.

[CR24] Floess S, Freyer J, Siewert C, Baron U, Olek S, Polansky J, Schlawe K, Chang H-D, Bopp T, Schmitt E (2007). Epigenetic control of the foxp3 locus in regulatory T cells. PLoS Biol.

[CR25] Fontenot JD, Gavin MA, Rudensky AY (2003). Foxp3 programs the development and function of CD4(+)CD25(+) regulatory T cells. Nat Immunol.

[CR26] Fontenot JD, Rasmussen JP, Gavin MA, Rudensky AY (2005). A function for interleukin 2 in Foxp3-expressing regulatory T cells. Nat Immunol..

[CR27] Hale JS, Fink PJ (2009). Back to the thymus: peripheral T cells come home. Immunol Cell Biol.

[CR28] Hanabuchi S, Ito T, Park WR, Watanabe N, Shaw JL, Roman E, Arima K, Wang YH, Voo KS, Cao W (2010). Thymic stromal lymphopoietin-activated plasmacytoid dendritic cells induce the generation of FOXP3+ regulatory T cells in human thymus. J Immunol.

[CR29] Hsieh CS, Liang Y, Tyznik AJ, Self SG, Liggitt D, Rudensky AY (2004). Recognition of the peripheral self by naturally arising CD25+ CD4+ T cell receptors. Immunity.

[CR30] Hu Z, Lancaster JN, Sasiponganan C, Ehrlich LI (2015). CCR4 promotes medullary entry and thymocyte-dendritic cell interactions required for central tolerance. J Exp Med.

[CR31] Josefowicz SZ, Lu LF, Rudensky AY (2012). Regulatory T cells: mechanisms of differentiation and function. Annu Rev Immunol.

[CR32] Josefowicz SZ, Niec RE, Kim HY, Treuting P, Chinen T, Zheng Y, Umetsu DT, Rudensky AY (2012). Extrathymically generated regulatory T cells control mucosal TH2 inflammation. Nature.

[CR33] Khailaie S, Robert PA, Toker A, Huehn J, Meyer-Hermann M (2014). A signal integration model of thymic selection and natural regulatory T cell commitment. J Immunol.

[CR34] Kieback E, Hilgenberg E, Stervbo U, Lampropoulou V, Shen P, Bunse M, Jaimes Y, Boudinot P, Radbruch A, Klemm U (2016). Thymus-derived regulatory T cells are positively selected on natural self-antigen through cognate interactions of high functional avidity. Immunity.

[CR35] Kim HJ, Cantor H (2011). Regulation of self-tolerance by Qa-1-restricted CD8(+) regulatory T cells. Sem Immunol.

[CR36] Kim HP, Leonard WJ (2007). CREB/ATF-dependent T cell receptor-induced FoxP3 gene expression: a role for DNA methylation. J Exp Med.

[CR37] Kisielow P, Miazek A (1995). Positive selection of T cells: rescue from programmed cell death and differentiation require continual engagement of the T cell receptor. J Exp Med.

[CR38] Kitagawa Y, Ohkura N, Kidani Y, Vandenbon A, Hirota K, Kawakami R, Yasuda K, Motooka D, Nakamura S, Kondo M (2017). Guidance of regulatory T cell development by Satb1-dependent super-enhancer establishment. Nature Immunol.

[CR39] Klein L, Jovanovic K (2011). Regulatory T cell lineage commitment in the thymus. Sem Immunol.

[CR40] Klein L, Hinterberger M, Wirnsberger G, Kyewski B (2009). Antigen presentation in the thymus for positive selection and central tolerance induction. Nat Rev Immunol.

[CR41] Konkel JE, Jin W, Abbatiello B, Grainger JR, Chen W (2014). Thymocyte apoptosis drives the intrathymic generation of regulatory T cells. Proc Natl Acad Sci USA.

[CR42] Kurd N, Robey EA (2016). T-cell selection in the thymus: a spatial and temporal perspective. Immunol Rev.

[CR43] Kyewski B, Klein L (2006). A central role for central tolerance. Annu Rev Immunol.

[CR44] Laufer TM, DeKoning J, Markowitz JS, Lo D, Glimcher LH (1996). Unopposed positive selection and autoreactivity in mice expressing class II MHC only on thymic cortex. Nature.

[CR45] Le Borgne M, Ladi E, Dzhagalov I, Herzmark P, Liao YF, Chakraborty AK, Robey EA (2009). The impact of negative selection on thymocyte migration in the medulla. Nat Immunol.

[CR46] Li MO, Sanjabi S, Flavell RA (2006). Transforming growth factor-beta controls development, homeostasis, and tolerance of T cells by regulatory T cell-dependent and -independent mechanisms. Immunity.

[CR47] Lio CW, Hsieh CS (2008). A two-step process for thymic regulatory T cell development. Immunity.

[CR48] Liston A, Nutsch KM, Farr AG, Lund JM, Rasmussen JP, Koni PA, Rudensky AY (2008). Differentiation of regulatory Foxp3+ T cells in the thymic cortex. Proc Natl Acad Sci USA.

[CR49] Liu Y, Zhang P, Li J, Kulkarni AB, Perruche S, Chen W (2008). A critical function for TGF-beta signaling in the development of natural CD4+CD25+Foxp3+ regulatory T cells. Nat Immunol.

[CR50] Love PE, Bhandoola A (2011). Signal integration and crosstalk during thymocyte migration and emigration. Nat Rev Immunol.

[CR51] Lucas B, McCarthy NI, Baik S, Cosway E, James KD, Parnell SM, White AJ, Jenkinson WE, Anderson G (2016). Control of the thymic medulla and its influence on alphabetaT-cell development. Immunol Rev.

[CR52] Mahmud SA, Manlove LS, Schmitz HM, Xing Y, Wang Y, Owen DL, Schenkel JM, Boomer JS, Green JM, Yagita H (2014). Costimulation via the tumor-necrosis factor receptor superfamily couples TCR signal strength to the thymic differentiation of regulatory T cells. Nat Immunol.

[CR53] Malchow S, Leventhal DS, Nishi S, Fischer BI, Shen L, Paner GP, Amit AS, Kang C, Geddes JE, Allison JP (2013). Aire-dependent thymic development of tumor-associated regulatory T cells. Science.

[CR54] Malek TR, Porter BO, Codias EK, Scibelli P, Yu A (2000). Normal lymphoid homeostasis and lack of lethal autoimmunity in mice containing mature T cells with severely impaired IL-2 receptors. J Immunol.

[CR55] Malek TR, Yu A, Vincek V, Scibelli P, Kong L (2002). CD4 regulatory T cells prevent lethal autoimmunity in IL-2Rbeta-deficient mice. Implications for the nonredundant function of IL-2. Immunity.

[CR56] Marie JC, Letterio JJ, Gavin M, Rudensky AY (2005). TGF-beta1 maintains suppressor function and Foxp3 expression in CD4+CD25+ regulatory T cells. J Exp Med.

[CR57] Marie JC, Liggitt D, Rudensky AY (2006). Cellular mechanisms of fatal early-onset autoimmunity in mice with the T cell-specific targeting of transforming growth factor-beta receptor. Immunity.

[CR58] Marshall D, Sinclair C, Tung S, Seddon B (2014). Differential requirement for IL-2 and IL-15 during bifurcated development of thymic regulatory T cells. J Immunol.

[CR59] Mazzucchelli R, Hixon JA, Spolski R, Chen X, Li WQ, Hall VL, Willette-Brown J, Hurwitz AA, Leonard WJ, Durum SK (2008). Development of regulatory T cells requires IL-7Ralpha stimulation by IL-7 or TSLP. Blood.

[CR60] McCaughtry TM, Wilken MS, Hogquist KA (2007). Thymic emigration revisited. J Exp Med.

[CR61] McCaughtry TM, Baldwin TA, Wilken MS, Hogquist KA (2008). Clonal deletion of thymocytes can occur in the cortex with no involvement of the medulla. J Exp Med.

[CR62] Meredith M, Zemmour D, Mathis D, Benoist C (2015). Aire controls gene expression in the thymic epithelium with ordered stochasticity. Nat Immunol.

[CR63] Ohigashi I, Kozai M, Takahama Y (2016). Development and developmental potential of cortical thymic epithelial cells. Immunol Rev.

[CR64] Ohkura N, Hamaguchi M, Morikawa H, Sugimura K, Tanaka A, Ito Y, Osaki M, Tanaka Y, Yamashita R, Nakano N (2012). T cell receptor stimulation-induced epigenetic changes and Foxp3 expression are independent and complementary events required for Treg cell development. Immunity.

[CR65] Ouyang W, Beckett O, Ma Q, Li MO (2010). Transforming growth factor-beta signaling curbs thymic negative selection promoting regulatory T cell development. Immunity.

[CR66] Pelly VS, Kannan Y, Coomes SM, Entwistle LJ, Ruckerl D, Seddon B, MacDonald AS, McKenzie A, Wilson MS (2016). IL-4-producing ILC2s are required for the differentiation of TH2 cells following Heligmosomoides polygyrus infection. Mucosal Immunol.

[CR67] Pennington DJ, Silva-Santos B, Silberzahn T, Escorcio-Correia M, Woodward MJ, Roberts SJ, Smith AL, Dyson PJ, Hayday AC (2006). Early events in the thymus affect the balance of effector and regulatory T cells. Nature.

[CR68] Popmihajlov Z, Xu D, Morgan H, Milligan Z, Smith KA (2012). Conditional IL-2 gene deletion: consequences for T cell proliferation. Front Immunol.

[CR69] Ribot J, Enault G, Pilipenko S, Huchenq A, Calise M, Hudrisier D, Romagnoli P, van Meerwijk JPM (2007). Shaping of the autoreactive regulatory T cell repertoire by thymic cortical positive selection. J Immunol.

[CR70] Romagnoli P, Hudrisier D, van Meerwijk JPM (2002). Preferential recognition of self-antigens despite normal thymic deletion of CD4+CD25+ regulatory T cells. J Immunol.

[CR71] Romagnoli P, Ribot J, Tellier J, van Meerwijk JPM, Jiang S (2008). Thymic and peripheral generation of CD4+Foxp3+ regulatory T cells. Regulatory T cells and clinical application.

[CR72] Sadlack B, Lohler J, Schorle H, Klebb G, Haber H, Sickel E, Noelle RJ, Horak I (1995). Generalized autoimmune disease in interleukin-2-deficient mice is triggered by an uncontrolled activation and proliferation of CD4+ T cells. Eur J Immunol.

[CR73] Sakaguchi S, Miyara M, Costantino CM, Hafler DA (2010). FOXP3+ regulatory T cells in the human immune system. Nat Rev Immunol.

[CR74] Shevach EM (2011). Biological functions of regulatory T cells. Adv Immunol.

[CR75] Shitara S, Hara T, Liang B, Wagatsuma K, Zuklys S, Hollander GA, Nakase H, Chiba T, Tani-ichi S, Ikuta K (2013). IL-7 produced by thymic epithelial cells plays a major role in the development of thymocytes and TCRgammadelta+ intraepithelial lymphocytes. J Immunol.

[CR76] Soper DM, Kasprowicz DJ, Ziegler SF (2007). IL-2Rbeta links IL-2R signaling with Foxp3 expression. Eur J Immunol.

[CR77] Stritesky GL, Xing Y, Erickson JR, Kalekar LA, Wang X, Mueller DL, Jameson SC, Hogquist KA (2013). Murine thymic selection quantified using a unique method to capture deleted T cells. Proc Natl Acad Sci USA.

[CR78] Sun J, Furio L, Mecheri R, van der Does AM, Lundeberg E, Saveanu L, Chen Y, van Endert P, Agerberth B, Diana J (2015). Pancreatic beta-cells limit autoimmune diabetes via an immunoregulatory antimicrobial peptide expressed under the influence of the gut microbiota. Immunity.

[CR200] Suzuki H, Kundig T, Furlonger C, Wakeham A, Timms E, Matsuyama T, Schmits R, Simard J, Ohashi P, Griesser H et al (1995) Deregulated T cell activation and autoimmunity in mice lacking interleukin-2 receptor beta. Science 268(5216):1472–1476 10.1126/science.7770771 7770771

[CR79] Suzuki H, Zhou YW, Kato M, Mak TW, Nakashima I (1999). Normal regulatory alpha/beta T cells effectively eliminate abnormally activated T cells lacking the interleukin 2 receptor beta in vivo. J Exp Med.

[CR80] Tai X, Cowan M, Feigenbaum L, Singer A (2005). CD28 costimulation of developing thymocytes induces Foxp3 expression and regulatory T cell differentiation independently of interleukin 2. Nat Immunol.

[CR81] Tai X, Erman B, Alag A, Mu J, Kimura M, Katz G, Guinter T, McCaughtry T, Etzensperger R, Feigenbaum L (2013). Foxp3 transcription factor is proapoptotic and lethal to developing regulatory T cells unless counterbalanced by cytokine survival signals. Immunity.

[CR82] Takaba H, Morishita Y, Tomofuji Y, Danks L, Nitta T, Komatsu N, Kodama T, Takayanagi H (2015). Fezf2 orchestrates a thymic program of self-antigen expression for immune tolerance. Cell.

[CR83] Tang Q, Henriksen KJ, Boden EK, Tooley AJ, Ye J, Subudhi SK, Zheng XX, Strom TB, Bluestone JA (2003). Cutting edge: CD28 controls peripheral homeostasis of CD4+CD25+ regulatory T cells. J Immunol.

[CR84] Tani-ichi S, Shimba A, Wagatsuma K, Miyachi H, Kitano S, Imai K, Hara T, Ikuta K (2013). Interleukin-7 receptor controls development and maturation of late stages of thymocyte subpopulations. Proc Natl Acad Sci USA.

[CR85] Thiault N, Darrigues J, Adoue V, Gros M, Binet B, Perals C, Leobon B, Fazilleau N, Joffre OP, Robey EA (2015). Peripheral regulatory T lymphocytes recirculating to the thymus suppress the development of their precursors. Nat Immunol.

[CR86] Toker A, Engelbert D, Garg G, Polansky JK, Floess S, Miyao T, Baron U, Duber S, Geffers R, Giehr P (2013). Active demethylation of the Foxp3 locus leads to the generation of stable regulatory T cells within the thymus. J Immunol.

[CR87] Ueno T, Saito F, Gray DH, Kuse S, Hieshima K, Nakano H, Kakiuchi T, Lipp M, Boyd RL, Takahama Y (2004). CCR7 signals are essential for cortex-medulla migration of developing thymocytes. J Exp Med.

[CR88] Valitutti S, Muller S, Cella M, Padovan E, Lanzavecchia A (1995). Serial triggering of many T-cell receptors by a few peptide-MHC complexes. Nature.

[CR89] Vang KB, Yang J, Mahmud SA, Burchill MA, Vegoe AL, Farrar MA (2008). IL-2, -7, and -15, but not thymic stromal lymphopoeitin, redundantly govern CD4+Foxp3+ regulatory T cell development. J Immunol.

[CR90] von Freeden-Jeffry U, Vieira P, Lucian LA, McNeil T, Burdach SE, Murray R (1995). Lymphopenia in interleukin (IL)-7 gene-deleted mice identifies IL-7 as a nonredundant cytokine. J Exp Med.

[CR91] Vuddamalay, Y., and van Meerwijk, J. (2017). CD28neg and CD28low CD8+ regulatory T cells: Of Mice and Men. Front Immunol 8. 10.3389/fimmu.2017.00031 PMC5256148 28167946

[CR92] Watanabe N, Wang YH, Lee HK, Ito T, Wang YH, Cao W, Liu YJ (2005). Hassall’s corpuscles instruct dendritic cells to induce CD4+CD25+ regulatory T cells in human thymus. Nature.

[CR93] Weist BM, Kurd N, Boussier J, Chan SW, Robey EA (2015). Thymic regulatory T cell niche size is dictated by limiting IL-2 from antigen-bearing dendritic cells and feedback competition. Nat Immunol.

[CR94] Wilkinson RW, Anderson G, Owen JJ, Jenkinson EJ (1995). Positive selection of thymocytes involves sustained interactions with the thymic microenvironment. J Immunol.

[CR201] Willerford DM, Chen J, Ferry JA, Davidson L, Ma A, Alt FW (1995) Interleukin-2 receptor α chain regulates the size and content of the peripheral lymphoid compartment. Immunity 3(4):521–530 10.1016/1074-7613(95)90180-9 7584142

[CR95] Wirnsberger G, Mair F, Klein L (2009). Regulatory T cells differentiation of thymocytes does not require a dedicated antigen-presenting cell but is under T cell-intrinsic developmental control. Proc Natl Acad Sci USA.

[CR96] Wolf M, Schimpl A, Hunig T (2001). Control of T cell hyperactivation in IL-2-deficient mice by CD4(+)CD25(-) and CD4(+)CD25(+) T cells: evidence for two distinct regulatory mechanisms. Eur J Immunol.

[CR97] Xu Z, Ho S, Chang CC, Zhang QY, Vasilescu ER, Vlad G, Suciu-Foca N (2016). Molecular and cellular characterization of human CD8 T suppressor cells. Front Immunol.

[CR98] Yamano T, Nedjic J, Hinterberger M, Steinert M, Koser S, Pinto S, Gerdes N, Lutgens E, Ishimaru N, Busslinger M (2015). Thymic B cells are licensed to present self antigens for central T cell tolerance induction. Immunity.

[CR99] Yang S, Fujikado N, Kolodin D, Benoist C, Mathis D (2015). Regulatory T cells generated early in life play a distinct role in maintaining self-tolerance. Science.

[CR100] Yao Z, Kanno Y, Kerenyi M, Stephens G, Durant L, Watford WT, Laurence A, Robinson GW, Shevach EM, Moriggl R (2007). Nonredundant roles for Stat5a/b in directly regulating Foxp3. Blood.

[CR101] Yu W, Nagaoka H, Jankovic M, Misulovin Z, Suh H, Rolink A, Melchers F, Meffre E, Nussenzweig MC (1999). Continued RAG expression in late stages of B cell development and no apparent re-induction after immunization. Nature.

[CR102] Yue X, Trifari S, Aijo T, Tsagaratou A, Pastor WA, Zepeda-Martinez JA, Lio CW, Li X, Huang Y, Vijayanand P (2016). Control of Foxp3 stability through modulation of TET activity. J Exp Med.

[CR103] Zheng Y, Josefowicz S, Chaudhry A, Peng XP, Forbush K, Rudensky AY (2010). Role of conserved non-coding DNA elements in the Foxp3 gene in regulatory T-cell fate. Nature.

